# Effects of metformin on endothelial function in type 2 diabetes

**DOI:** 10.3892/etm.2014.1582

**Published:** 2014-02-24

**Authors:** SONGLIN WU, XIAOYAN LI, HONGMING ZHANG

**Affiliations:** 1 Department of Geriatrics, Renmin Hospital of Wuhan University, Wuhan, Hubei 430060, P.R. China; 2 Department of Cardiology, Jinan Military General Hospital, Jinan, Shandong 250031, P.R. China

**Keywords:** metformin, type 2 diabetes mellitus, vascular endothelial function

## Abstract

The aim of the present study was to examine the effect of metformin on endothelial function in patients with type 2 diabetes mellitus (T2DM). In total, 93 patients with T2DM and dissatisfactory glycemic control were randomly assigned to the metformin and pioglitazone groups and changes in vascular endothelial function were subsequently observed. Blood sugar levels and the insulin resistance (IR) index of the patients prior to treatment were lower than those following 12 months of treatment. In addition, fasting and postprandial insulin levels and the insulin function index were higher compared with those obtained following 12 months of treatment (P<0.05). Following 12 months of treatment, the body mass index (BMI) in the metformin group was lower than that in the pioglitazone group (P<0.05). Vascular endothelial function had improved in the groups following 12 months of treatment, when compared with the levels prior to treatment (P<0.05). Following 12 months of treatment, endothelial function in the metformin group had improved markedly compared with that in the pioglitazone group (P<0.05). Therefore, the administration of metformin and pioglitazone in patients with T2DM may improve insulin function, reduce the role of IR and improve endothelial function. Metformin is more successful than pioglitazone in reducing BMI and improving endothelial function.

## Introduction

Diabetes mellitus (DM) is a common endocrine disease. Various acute and chronic complications of DM are serious threats to human health. Vascular disease is the most important complication of DM and underlies other chronic complications. The pathogenesis of DM vascular lesions is relative to vascular endothelial cell injury, platelet activation and the abnormality of coagulation and fibrinolysis (1). Endothelial cell injury is a vital early manifestation of DM vascular disease and is the initiating factor in the development of atherosclerosis (2,3). Therefore, the improvement of vascular endothelial function is crucial for the treatment of DM. The effects of metformin on vascular endothelial function had not been reported previously, thus, the present study was undertaken with the aim of investigating the effects of metformin on vascular endothelial function in patients with type 2 DM (T2DM).

## Materials and methods

### Subjects

A total of 93 patients were selected for this study. The individuals were treated with a diabetes diet, exercise and hypoglycemic drugs (without the use of biguanides and thiazolidinediones) and had fasting blood glucose (FBG) levels of >7.8 mmol/l and/or 2 h postprandial blood glucose (2hPBG) levels of >10.0 mmol/l. The patients were admitted to Renmin Hospital of Wuhan University (Wuhan, China) between September 2010 and August 2012 and comprised 48 males and 45 females with a mean age of 60.3±9.7 years and a mean disease course length of 6.1±5.6 years. All the patients were diagnosed with T2DM according to the World Health Organization’s diagnostic criteria (1999) (4). Patients with T1DM, hypertension, hyperlipidemia, kidney disease, infection, heart failure, thyroid dysfunction, diabetic ketoacidosis and those who smoked were excluded from this study. All participants signed an informed consent form. The study was approved by the Ethics Committee of the Renmin Hospital of Wuhan University.

### Methods

A total of 93 patients were randomly divided into the metformin (500 mg 3 times/day; Sino-american Shanghai squibb Co., Ltd., Shanghai, China) and pioglitazone groups [15 mg once/day; Takeda Pharmaceutical (China) Company Limited, Tianjin, China]. All the patients were treated with drugs combined with diet control and exercise for 12 months. General clinical data, including gender, age, body mass index (BMI), disease course and blood sugar levels, of the subjects were obtained prior to treatment (Table I).

### Determination of glucose, insulin and islet function

FBG and 2hPBG levels were measured using the hexokinase method (Mairui BS300 Biochemical analyzer; Shenzhen Mairui Biological Medical Electronic Co., Ltd., Shenzhen, China). Fasting insulin (FIns) [ADVIA Centaur XP, Siemens (China) Co., Ltd., Shenzhen, China] and glycosylated hemoglobin (HbA1C) (Variant II Hemoglobin A1cProgram, Bio-Rad, Hercules, CA, USA) levels were measured by radioimmunoassay (RIA) and immunoturbidimetry, respectively. The insulin resistance (IR) index of the homeostasis model was calculated using the following formula: FIns × FBG/22.5. Pancreatic β cell function was calculated using the following formula: FIns × 20/(FBG − 3.5) (5).

### Detection of vascular endothelial function

According to the method by Celermajer *et al* (6), vascular endothelial function was detected by observing brachial artery expansion using high resolution ultrasound. This was detected using an ultrasonic diagnosis instrument (HP-2500, Hewlett-Packard Company, Palo Alto, CA, USA) after the patients had ceased to use the vasodilator for >24 h and had fasted for 12 h. Subjects were placed in the supine position and allowed to rest for at least 10 min. A 7.5 MHz linear probe with a probing depth of 4 cm was used and an electrocardiogram was recorded at the same time. The right upper limb abduced by 15° with the palm facing upwards. The 2–15 cm brachial artery above elbow was selected, a long axis view of the brachial artery was exhibited and the end diastolic inner-diameter was measured. The diameter was measured over three heartbeat cycles and the average values were calculated. The diameter of the brachial artery of each subject was measured at rest, during reactive hyperemia and rest following the administration of nitroglycerin. Firstly, the inner-diameter of the brachial artery at rest was measured (D_0_). Subsequently, a sphygmomanometer cuff was tied 2–3 cm below the elbow and the systolic blood pressure was maintained at >50 mmHg by pneumatic compression and was deflated after being maintained for 4–5 min. The inner-diameter of the brachial artery following reactive hyperemia was measured within 60–90 sec of deflation (D_1_). Subjects rested for 5 min and were administered 0.5 mg nitroglycerin sublingually after the vessel had returned to normal; the inner-diameter of the brachial artery was measured 3–4 min later (D_2_). The probe was always in a fixed position, the same technique parameters were used throughout the whole process and the vessel diameter was measured at the same site each time. The percentage of vascular dilatation was calculated using the following formulas: Vasodilation rates after reactive hyperemia (%) = (D1−D0)/D0 × 100, and Vasodilation rates after taking nitroglycerin (%) = (D2−D0)/D0 × 100. Results that were >10% were considered to be normal and those <10% were considered abnormal.

### Detection of plasma endothelin (ET-1) and serum nitric oxide (NO)

Plasma ET-1 levels were measured using the nitrate reductase method with an ET-1 ELISA kit (Enzyme-linked biological technology Co., Ltd., Shanghai, China), according to the manufacturer’s instructions. Serum NO levels were also measured using the nitrate reductase method with an NO kit according to the manufacturer’s instructions (Enzyme-linked biological technology Co., Ltd.).

### Observation of adverse reactions and liver and kidney function

Routine blood tests and liver and kidney function analyses were performed every three months prior to and following treatment. The adverse reactions of the drugs were recorded.

### Statistical analysis

Statistical analysis was performed using Statistical Package for the Social Science (SPSS version 13.0; SPSS, Inc., Chicago, IL, USA). P<0.05 was considered to indicate a statistically significant difference. Measurement data with a normal distribution were compared using analysis of variance among multi-groups and a t-test between two groups. The χ^2^ test was used to compare rates, while the rank-sum test was used to compare data with a non normal distribution.

## Results

### General data

There were no statistically significant differences in the general clinical data, including gender, age, BMI, disease course and blood sugar levels, between the two groups prior to treatment (P>0.05; Table I).

### Hypoglycemic situation

There was no significant difference between the two groups receiving treatment combined with other antidiabetic drugs (P>0.05; Table II). The FBG, 2hPBG and HbA1C levels of the metformin and pioglitazone groups were significantly lower following 12 months of therapy compared with the values obtained prior to the administration of therapy (P<0.01). There were no significant differences in the FBG, 2hPBG and HbA1C levels between the two groups following treatment (P>0.05; Table III).

### BMI

Following 12 months of treatment, the BMI of the metformin group was significantly lower than that calculated prior to therapy (P<0.05). There was no significant difference in the BMI of the pioglitazone group prior to and following treatment. The BMI of the metformin group was significantly lower than that of the pioglitazone group (P<0.05; Tables III and IV).

### Islet function and IR index

Following 12 months of treatment, the FIns, pancreatic β cell function and IR index of the patients in the metformin and pioglitazone groups were significantly lower than that measured prior to therapy (P<0.05). However, there was no statistically significant difference between the metformin and pioglitazone groups (P>0.05; Tables III and IV).

### Vascular endothelial function

Following 12 months of treatment, the vascular dilation rates of the patients in the metformin and pioglitazone groups during reactive hyperemia and following the administration of nitroglycerin significantly increased compared with that measured prior to therapy (P<0.05). The improvement of vascular endothelial function in the metformin group was greater than that of the pioglitazone group (P<0.05; Tables III and IV).

### ET-1 and NO levels

Analysis of ET-1 and NO levels revealed that following 12 months of treatment, the ET-1 levels of the two groups decreased significantly compared with the levels prior to treatment (P<0.05). However, the NO levels of the two groups increased significantly compared with the levels prior to therapy (P<0.05; Table V). The improvement in vascular endothelial function in the metformin group was greater than that in the pioglitazone group (P<0.05; Table VI).

### Adverse reaction

There were five patients in the metformin group who exhibited varying degrees of nausea and epigastric discomfort, however, these individuals did not present with vomiting, diarrhea or any other symptoms. There were four patients in the pioglitazone group who presented with lower extremity edema. No abnormal liver and renal function was observed or any other adverse reactions in the two groups.

## Disscussion

Biguanides launched as a treatment for DM in the late 1950s and mainly included phenformin and metformin. Shortly thereafter, lactic acidosis associated with biguanides was paid increasing attention. In 1968, the University Group Diabetes Program reported that phenformin increased the mortality rate of cardiovascular disease, thus, the use of biguanides in clinical practice decreased (7). In 1998, the UK Prospective Diabetes Study confirmed that metformin was not only a hypoglycemic drug capable of reducing macrovascular complications, but it was also capable of reducing the incidence of diabetic complications and diabetic patient mortality. Thus, metformin became a first-line hypoglycemic drug and was studied further (8).

In the present study, vascular endothelial function was detected by observing brachial artery expansion using high resolution ultrasound. This non-invasive method was pioneered by Celermajer *et al* (6) in 1992. The results of the present study confirmed that following 12 months of treatment, the vascular dilation rates of the metformin and pioglitazone groups in reactive hyperemia and following the administration of nitroglycerin significantly increased when compared with the rates observed prior to therapy (P<0.05). Vascular endothelial function in the metformin group exhibited a greater level of improvement compared with that of the pioglitazone group (P<0.05). The detection of ET-1 and NO levels demonstrated that following 12 months of treatment, the ET-1 levels of the two groups decreased significantly compared with the levels measured prior to therapy (P<0.05), while the NO levels of the two groups increased significantly compared with those measured prior to therapy (P<0.05). Vascular endothelial function in the metformin group demonstrated a greater level of improvement compared with that of the pioglitazone group (P<0.05). These results were consistent with the ultrasound observations of brachial artery endothelial function and demonstrated that metformin and pioglitazone were capable of improving the vascular endothelial function of diabetic patients, yet metformin was more successful than pioglitazone.

The results of the present study demonstrated that following 12 months of treatment the FIns levels, pancreatic β cell function and IR index of the metformin and pioglitazone groups were significantly lower than that measured prior to therapy (P<0.05); there was no statistical difference between the two groups (P>0.05). Thus, the results indicate that metformin and pioglitazone are capable of improving islet function and reducing the IR index with similar curative effects.

Hyperglycemia may damage the vascular endothelium by oxidative stress. It has been confirmed that oxidative stress is key in the development of macrovascular and microvascular complications in diabetic patients. An early sign of vascular injury caused by oxidative stress is endothelial dysfunction (9). In addition, advanced glycation end-products of vascular endothelial cells can mediate oxygen free radicals by binding with a receptor in the vascular endothelial cells directly and/or indirectly, impairing vascular endothelial function (10). Additionally, IR may also lead to the abnormal function of vascular endothelial cells (11). Therefore, we hypothesize that metformin and pioglitazone improve endothelial function by reducing blood sugar levels, improving islet function and reducing the IR index. The present study also confirmed that metformin and pioglitazone have a similar curative effect in reducing blood sugar levels, improving islet function and reducing the IR index. However, metformin is more successful at improving endothelial function than pioglitazone, the reason for which is currently unclear and requires further study.

## Figures and Tables

**Table I:** Comparison of general clinical data between the two groups prior to treatment.

Group	Cases, n	Age, years	Gender, male, n (%)	Course length, years	Blood glucose, mmol/l		HbA1C, %	BMI, kg/m^2^

					FBG	2hPBG		
Metformin	47	60.1±9.6	17 (50)	7.3	8.0±1.6	12.82±3.7	7.2±2.4	24.9±5.8
Pioglitazone	46	60.3±9.7	17 (51.5)	7.2	8.1±1.6	12.69±3.6	7.1±2.5	24.6±5.6
P-value		0.183	0.412	0.089	0.215	0.176	0.343	0.358

BMI, body mass index; HbA1C, glycosylated hemoglobin; FBG, fasting blood glucose; 2hPBG, 2 h postprandial blood glucose.

**Table II:** Comparison of treatment combined with other antidiabetic drugs in the two groups.

Group	Cases, n	Sulfonylurea cases, n (%)	Non-sulfonylurea insulin secretagogues cases, n (%)	Glucosidase inhibitors cases, n (%)	Insulin cases, n (%)
Metformin	47	12 (25.53)	10 (21.28)	26 (55.32)	13 (27.66)
Pioglitazone	46	11 (23.91)	9 (19.57)	25 (54.35)	12 (26.09)
P-value		0.316	0.094	0.512	0.185

**Table III:** Comparison of blood glucose, body mass index and islet function in two groups respectively prior to and following treatment.

Group	Blood glucose, mmol/l		HbA1C, %	BMI, kg/m^2^	FIns, mU/l	IR index	Pancreatic β cell function	Vascular dilation rates, %	
	
	FBG	2hPBG						Reactive hyperemia	After nitroglycerin
Metformin (n=47)									
Prior to treatment	8.0±1.6	12.8±3.7	7.2±2.4	25.6±5.8	17.4±5.2	7.7±1.6	66.6±8.5	8.5±2.2	15.8±4.3
Following treatment	6.3±1.6	8.9±3.1	6.3±2.2	22.1±5.5	20.2±5.5	6.3±1.4	112.2±13.7	11.3±3.1	17.1±4.5
P-value	0.008	0.004	0.007	0.042	0.032	0.043	0.036	0.037	0.041
Pioglitazone (n=46)									
Prior to treatment	8.1±1.6	12.7±3.6	7.1±2.5	25.4±5.7	17.2±5.3	7.8±1.7	68.0±8.8	8.7±2.3	14.4±4.2
Following treatment	6.4±1.6	9.1±3.2	6.4±2.3	24.8±5.6	19.9±5.3	6.1±1.3	119.8±14.3	9.2±2.4	15.7±4.3
P-value	0.007	0.009	0.006	0.087	0.027	0.019	0.017	0.021	0.011

Values are expressed as mean ± SD. BMI, body mass index; HbA1C, glycosylated hemoglobin; FBG, fasting blood glucose; 2hPBG, 2 h postprandial blood glucose; FIns, fasting insulin; IR, insulin resistance.

**Table IV:** Comparison of blood glucose, BMI and islet function between the two groups following treatment.

Groups	Blood glucose, mmol/l		HbA1C, %	BMI, kg/m^2^	FIns, mU/l	IR index	Pancreatic β cell function	Vascular dilation rates, %	
	
	FBG	2hPBG						Reactive hyperemia	After nitroglycerin
Metformin (n=47)	6.3±1.6	8.9±3.1	6.3±2.2	22.1±5.5	20.2±5.5	6.3±1.4	112.2±13.7	11.3±3.1	17.1±4.5
Pioglitazone (n=46)	6.4±1.6	9.1±3.2	6.4±2.3	24.8±5.6	19.9±5.3	6.1±1.3	119.8±14.3	9.2±2.4	15.7±4.3
P-value	0.241	0.228	0.361	0.037	0.378	0.328	0.292	0.026	0.019

Values are expressed as mean ± SD. BMI, body mass index; HbA1C, glycosylated hemoglobin; FBG, fasting blood glucose; 2hPBG, 2 h postprandial blood glucose; FIns, fasting insulin; IR, insulin resistance.

**Table V:** Comparison of NO and ET-1 levels in the two groups, prior to and following treatment.

Group	ET-1, ng/l	NO, μmol/l
Metformin (n=47)		
Prior to treatment	81.3±12.8	35.6±8.3
Following treatment	37.5±7.4	60.3±11.2
P-value	0.008	0.009
Pioglitazone (n=46)		
Prior to treatment	82.2±12.6	34.7±9.0
Following treatment	57.6±9.4	50.1±10.4
P-value	0.030	0.042

Values are expressed as mean ± SD. ET-1, endothelin; NO, nitric oxide.

**Table VI:** Comparison of NO and ET-1 levels between the two groups following treatment.

Group	ET-1, ng/l	NO, μmol/l
Metformin (n=47)	37.5±7.4	60.3±11.2
Pioglitazone (n=46)	57.6±9.4	50.1±10.4
P-value	0.041	0.045

Values are expressed as mean ± SD. ET-1, endothelin; NO, nitric oxide.
